# Correlates of uptake of HIV testing among children and young adolescents in Akwa-Ibom state, Nigeria: a secondary data analysis of the Akwa-Ibom aids indicator survey, 2017

**DOI:** 10.1186/s12887-021-02495-5

**Published:** 2021-01-12

**Authors:** Damilola Adetoro, Hadiza Khamofu, Titilope Badru, John Markson, Oluwasanmi Adedokun, Nana Sandah-Abubakar, Ibrahim Dafa, Mario Chen, Robert Chiegil, Kwasi Torpey

**Affiliations:** 1 University of Ghana School of Public Health, Accra, Ghana; 2 FHI 360 Nigeria, Abuja, Nigeria; 3 Akwa Ibom State Government, Uyo, Nigeria; 4 National Primary Healthcare Development Agency, Abuja, Nigeria; 5 Achieving Health Nigeria Initiative, Abuja, Nigeria; 6 FHI360, Durham, North Carolina USA

**Keywords:** Children, Young adolescents, Caregivers, HIV, HIV testing, AIDS, Early infant diagnosis, Nigeria

## Abstract

**Background:**

In order to end the AIDS epidemic by 2030, there is a need to significantly reduce the rate of new infection among children and young adolescents. Identifying the correlates of testing behaviour is necessary to improve HIV testing campaigns by refining messages that target individuals in this age group. The objective of this study was to determine the correlates of HIV testing among children and young adolescents in Akwa-Ibom, Nigeria.

**Methods:**

The outcome was a secondary data analysis of the 2017 Akwa-Ibom AIDS Indicator Survey. Data of 4037 children and young adolescents aged 0–14 years was assessed in this study. Analysis was done using STATA version 16. Chi-squared test and logistic regression models were used to measure association and its strength between uptake of HIV testing and some independent variables (child/caregiver’s age, sex, educational status, child’s location, caregiver’s knowledge of HIV and caregiver ever tested for HIV) at 5% significance level.

**Results:**

Result showed that only 14.2% of the children and young adolescents have been tested for HIV. Previous history of blood transfusion (AOR = 5.33, 95%C.I = 2.60–10.92, *P* = < 0.001), caregiver’s level of education (AOR = 2.67, 95%C.I = 1.30–5.51, *P* = 0.008) and caregiver ever tested for HIV (AOR = 8.31, 95%C.I = 5.67–12.19, *P* = < 0.001) were significantly associated with uptake of HIV testing.

**Conclusion:**

This study concludes that a large proportion of children and young adolescents in Akwa-Ibom state have never been tested for HIV. There is a need for HIV testing interventions to be targeted towards this age groups and their parents/guardian. Addressing the knowledge gap amongst caregivers especially in rural areas is crucial towards improving the effectiveness of HIV testing interventions.

**Supplementary Information:**

The online version contains supplementary material available at 10.1186/s12887-021-02495-5.

## Background

HIV remains one of the public health challenges of our time; responsible for morbidity and mortality across different age group [1]. It is estimated that there are about 37,900,000 people living with HIV globally [2]. Of these, 36.2 million were adults and 1.7 million were children less than 15 years old [2]. According to the Joint United Nations Programme on HIV/AIDS (UNAIDS), an estimated 1.7 million individuals worldwide became newly infected with HIV in 2018; 160,000 new infections among children ages 0–14 [3]. In 2018, only 79% of people with HIV globally knew their HIV status [2]. Although new HIV infections have declined by an estimated 16%, from 2.1 million in 2010 to 1.7 million in 2018, around 770,000 deaths were recorded from AIDS-related illnesses worldwide in 2018 [2].

Globally, mother-to-child transmission of HIV accounts for more than 90% of all new paediatric HIV infections and may occur during pregnancy, labor, delivery or breastfeeding [4]. The risk of mother to child transmission during pregnancy ranges from 20 to 45%. However, with specific interventions, this risk can be reduced to less than 2% [5]. Without testing and treatment, 50% of HIV-positive infants die by age 2 years and 75% by age 5 years. For children who became infected through vertical transmission or other means such as unsafe blood transfusions, the best protection against dying from AIDS is HIV testing and diagnosis followed by prompt ARV treatment [6].

About 1.9 million Nigerians are living with HIV, giving a national prevalence of 1.4% [7]. This makes Nigeria the country with the second highest burden of HIV globally [8]. The Nigeria HIV/AIDS Indicator and Impact survey in 2018 estimated the prevalence among children aged 0–14 years to be 0.2%. HIV prevalence varied by zone across Nigeria, with the highest in South-South Zone (3.1%) and Akwa-Ibom state recording the highest in the country- 5.5% [7]. Reports from the Nigeria HIV/AIDS Indicator and Impact Survey (NAIIS) also showed that only 40% of women who gave birth since January 1, 2015 knew their HIV status despite the investment in safe and cost effective prevention of mother to child transmission (PMTCT) interventions for HIV in the country [7]. This limits the identification of HIV exposed children and proportion of those accessing HIV testing services which is the basis for timely initiation of antiretroviral therapy [9]. As a PMTCT priority state [10], the identification of correlates of testing behaviour is extremely important. This can be used to improve HIV testing campaigns by refining messages that target individuals at highest risk of infection [11].

Both individual and institutional factors have been found to affect uptake of testing services in sub Saharan Africa [12]. Individual factors were mainly related to parents or guardians of the children and included demographic (e.g., gender, age, marital status) and socioeconomic factors (e.g., income, urban/rural residence, education attainment) [12–15]. The institutional factors were mainly inadequate human resources, supplies and weak infrastructure [12, 16].

Given that there is still no cure for HIV, prevention continues to be a main thrust of HIV control efforts. HIV testing and counselling is central to these efforts. It presents valuable opportunities to identify new infections, increase awareness of HIV status, and addresses the HIV related health disparities [17]. In addition, it alleviates anxiety, promotes behavioural change and facilitates early referral to care and support, including access to antiretroviral (ARV) drug therapy [18]. By implementing focused, high-impact prevention and accelerated HIV testing, it is expected that 95% of people living with HIV should know their HIV status by 2030 [19]. This would mark the end of the AIDS epidemic as a public health threat and reduce drastically the number of people acquiring new HIV infection [20–22].

Many studies have assessed knowledge, perception and factors influencing HIV testing among adult but there is a dearth of studies on the correlates of factors influencing testing amongst children and young adolescents. This will help inform public health policy to develop a more holistically targeted and context specific HIV testing program for children and young adolescents as an important HIV prevention and treatment strategy. Such knowledge will also help combat the factors that may mitigate uptake of testing services [23]. Ending the AIDS epidemic is an opportunity to lay the foundation for a healthier world for future generations [24]. It will be impossible to end the epidemic without bringing HIV treatment to all who need it. Testing helps identify those to be treated. This study therefore aims to determine the correlates of HIV testing amongst children and young adolescents in Akwa-Ibom State, Nigeria using data collected as part of the Akwa-Ibom AIDS indicator survey (AKAIS) (a population based cross-sectional survey that offered household-based HIV testing, conducted between April and June 2017).

## Methods

### Survey methodology

This study is based on a secondary data analysis from the Akwa-Ibom AIDS Indicator Survey (AKAIS). The AKAIS was a population-based cross-sectional survey amongst 15,609 household residents across all 31 local government areas (LGAs) of Akwa-Ibom state between April and June 2017. The AKAIS sample was a stratified multi-stage sample with two stages. In the first stage, 226 enumeration areas (EA) were selected using probability proportional to size (PPS). The AKAIS used enumeration areas and population information defined by the 2006 population census. In the second stage of sampling, a fixed number of households were sampled from each selected EA using systematic sampling. This was done following a household mapping and enumeration exercise to update the EA sampling weights.

The Centers for Disease Control (CDC) questionnaires to guide HIV Impact Assessment (HIA) surveys was adapted for the AKAIS. Parental consent was sought from the parent or guardian of children 0–14 years. In addition, assent was sought from children aged 10–14 years whose parent or guardian had consented to their participation. Individual records were uniquely identified using barcode identification numbers. Questions pertaining to children 0 months–14 years were embedded in the individual questionnaire for adults, and data was provided by their parent or guardian (Supplementary file 1). A separate questionnaire for adolescents ages 10–14 was administered to eligible participants in this age group. Information was collected on demographic characteristics; HIV knowledge, attitudes, and risk perception; HIV testing; Adolescents aged 12–14 years were asked additional questions about sexual activity (Supplementary file 2).

### Study measures

The sample for this study were all 4037 children and adolescents aged 0–14 years whose caregivers answered the question “ever tested for HIV?” Data for 2167 caregivers was analyzed.

Uptake of HIV testing amongst children and adolescent was the outcome variable for this study. It was assessed by asking caregiver of children and adolescent the question: ‘has child ever tested for HIV?’ It was measured as a categorical variable and assessed as a binary outcome – yes and no. Child characteristics included age, sex, child enrolled in school, location, child ever received blood transfusion while caregiver-related characteristics analyzed were age, sex, educational status, comprehensive knowledge of HIV and caregiver ever tested for HIV. Caregivers comprehensive knowledge of HIV was assessed, and this was defined as: i) knowing that someone can protect himself/herself from HIV by using condom during sexual intercourse, ii) knowing that a healthy-looking person can have HIV, iii) knowing that HIV can be transmitted by having unprotected sex with an HIV-infected person, iv) knowing that there are medicines that people with HIV can take to help them live longer, and v) knowing that HIV can be transmitted by sharing of sharp objects. A binary outcome of “1” was designated if all questions were answered correctly and “0” if any of the questions were answered incorrectly.

### Statistical analysis

Statistical analysis was performed using Stata/SE 16.0, and the ‘svy’ command was used to account for the complex survey design including clustering. The analysis was done adjusting for sampling weights, hence, weighted estimates were reported. A subpopulation analysis for children and adolescent was applied. Descriptive statistics were used to provide characteristics of the study population.

Design adjusted Chi square test of association was conducted to explore the empirical relationship between uptake of HIV testing and selected independent variables which include child’s age, sex, location, enrolment in school, ever transfused with blood, caregiver’s age, caregiver’s sex, caregiver’s highest level of education, comprehensive knowledge of HIV and caregiver ever tested for HIV.

Design adjusted simple and multiple logistic regressions were used to estimate crude and adjusted odds ratio. Variables significant in the bivariate analysis (*P* < 0.05) were included in the multivariable logistic regression model. However, insignificant variables that are of contextual importance such as child’s enrollment in school, sex of the caregiver, and sex of the child were included in the multivariable logistic regression model. The goodness of fit of the model was assessed using the Hosmer-Lemeshow test. Odds ratio were used to assess the strength of association between the independent factors and uptake of HIV testing. *P*-values less than 0.05 were considered statistically significant.

## Results

### Socio-demographic characteristics of participants

The mean age of the children was 6.20 yrs. ± 3.91. Less than a quarter (23.3%) of them were young adolescents between the ages of 10–14 years while 76.7% were children less than 10 years. Approximately equal numbers were male (50.8%) and female (49.2%). 31.8% of the respondents lived in urban areas. A quarter of the children (26.2%) were not enrolled in school. Majority of children (98.4%) have never received blood transfusion (Table 1).

**Table 1:** Socio-demographic characteristics of participants

Variable	Frequency (unweighted) ***N*** = 4037	Weighted % or Mean
**Child characteristics**		
**Age (years)**		
< 1	275	6.86
1–4	1340	33.09
5–9	1477	36.71
10–14	945	23.34
**Sex**		
Male	2048	50.76
Female	1989	49.24
**Location**		
Urban	1247	31.77
Rural	2790	68.23
**Ever transfused with blood**		
No	3975	98.44
Yes	62	1.56
**Child enrolled in school**		
No	1058	26.20
Yes	2979	73.80
**Child ever tested for HIV**		
No	3469	85.85
Yes	568	14.15
**Caregiver characteristics**		
**Age (years)**		
15–24	402	9.90
25–34	1404	34.83
35–44	1266	31.14
≥ 45	965	24.13
**Sex**		
Male	1441	35.58
Female	2596	64.42
**Educational status**		
None	358	8.64
Primary	1596	39.13
Secondary	1674	41.86
Tertiary	407	10.37
**Comprehensive knowledge of HIV**		
No	2893	72.12
Yes	1144	27.88
**Caregiver ever tested for HIV**		
No	1847	45.69
Yes	2190	54.31

*%* Percentage

Only 14.2% (95% C.I = 12.49–15.99) of children and young adolescents reported ever testing for HIV. Majority (85.9%) of the participants have never been tested.

### Caregiver/parent factors

Caregiver/parent factors consisted of the following variables: age, sex, highest level of education, comprehensive knowledge of HIV and ever tested for HIV.

The mean age of the caregivers/parents was 37.46 ± 11.62. 64.4% were females. Approximately one-tenth (8.6%) had no form of education; two-fifths (41.9%) had secondary education while only 10.4% had a tertiary education. Less than a third (27.9%) of the caregivers had a comprehensive knowledge of HIV while slightly above half (54.3%) had ever been tested for HIV (Table 1).

### Association between selected factors and uptake of HIV testing

Table 2 presents the bivariate analysis of selected independent variables and uptake of HIV testing. On average, 15.0% of children less than 10 years and 10.4% of adolescents between 10 and 14 years had ever been tested for HIV (*p* < 0.001). An increase in the age of the children (> 4 years) was associated with a reduction in uptake of HIV testing. Only 12.2% of rural dwellers had been tested as compared to 18.3% of urban dwellers (*p* = 0.002). A higher proportion of children who have ever received blood transfusion have ever been tested for HIV compared to those who had never received blood transfusion (46.1% vs. 13.6%, *p* < 0.001).

**Table 2:** Bivariate and multivariate analysis of factors associated with ever being tested for HIV among children and young adolescents in Akwa-Ibom state

	Ever tested for HIV			
	Bivariate analysis		Multivariate analysis	
	n (%)	***P*** value	AOR (95% C.I)^**a**^	***P*** value
**Child characteristics**				
**Age (years)**				
< 1	40 (14.14)		1	
1–4	229 (17.20)	< 0.001*	1.22 (0.83–1.81)	0.308
5–9	201 (13.79)		0.94 (0.60–1.47)	0.784
10–14	98 (10.39)		0.72 (0.44–1.18)	0.187
**Sex**				
Male	286 (14.09)		1	
Female	282 (14.21)	0.910	0.96 (0.79–1.17)	0.709
**Location**				
Rural	341 (12.20)		1	
Urban	227 (18.33)	0.002*	1.11 (0.82–1.51)	0.500
**Ever transfused with blood**				
No	538 (13.64)		1	
Yes	30 (46.11)	< 0.001*	5.33 (2.60–10.92)	< 0.001*
**Enrolled in school**				
No	152 (14.29)		1	
yes	416 (14.10)	0.882	1.11 (0.83–1.49)	0.484
**Caregiver characteristics**				
**Age (years)**				
15–24	51 (12.75)		1	
25–34	228 (16.40)	< 0.001*	1.08 (0.72–1.61)	0.700
35–44	203 (16.30)		1.37 (0.87–2.15)	0.172
≥ 45	86 (8.70)		0.78 (0.46–1.33)	0.356
**Sex**				
Male	189 (12.79)		1	
Female	379 (14.90)	0.187	0.97 (0.71–1.31)	0.823
**Educational status**				
None	16 (4.44)		1	
Primary	195 (12.03)	< 0.001*	2.22 (1.04–4.75)	0.039*
Secondary	253 (15.33)		2.09 (1.00–4.41)	0.054
Tertiary	104 (25.52)		2.67 (1.30–5.51)	0.008*
**Comprehensive knowledge of HIV**				
No	355 (12.19)		1	
Yes	213 (19.21)	< 0.001*	1.22 (0.94–1.58)	0.133
**Caregiver ever tested for HIV**				
No	57 (3.10)		1	
yes	511 (23.45)	< 0.001*	8.31 (5.67–12.19)	< 0.001*

*n(%)* Unweighted frequency (weighted percentage), *C.I* Confidence interval, *AOR* Adjusted odds ratio

*Significant (*P* < 0.05)

^a^Measure of the strength of association between selected factors and uptake of HIV testing

### Association between caregiver factors and uptake of HIV testing

As shown in Table 2, 12.8, 16.4, 16.3, 8.7% of children whose caregivers were 15–24 years, 25–34 years, 35–44 years, ≥45 years, respectively, had ever tested for HIV (*p* < 0.001). Increasing age of caregiver (> 35 years) was associated with a reduction in uptake of HIV testing among the children. Uptake of HIV testing was only slightly higher amongst children whose caregivers were females (14.9%) as compared to males (12.8%). Prior HIV testing was highest among caregivers with tertiary education (25.5%) compared to those without education (4.4%), primary (12.0%), and secondary education (15.3%) (*p* < 0.001). An increase in caregiver’s educational status was associated with an increase in uptake of HIV testing among the participants. A higher proportion of children whose caregivers have comprehensive knowledge of HIV (19.2%) had ever tested for HIV than children whose caregivers have no comprehensive knowledge of HIV (12.2%) (*p* < 0.001).

### Multivariate analysis of correlates of uptake of HIV testing

Table 2 presents information on the multivariable logistic regression of correlates of uptake of HIV testing. The multivariable logistic regression revealed previous history of transfusion, caregiver’s highest level of education and caregiver ever tested for HIV as significant factors associated with uptake of HIV testing in children and young adolescents.

Children with a previous history of blood transfusion were 5.3 times (AOR = 5.33, 95%CI = 2.60–10.92) more likely to have been tested after adjusting for other factors. Those whose caregivers’ highest level of education was primary education were 2.2 times (AOR = 2.22, 95%C.I = 1.04–4.75) more likely to have been tested compared to those with no education. This was slightly higher for those whose caregivers’ highest level of education was tertiary (AOR = 2.67, 95%CI = 1.30–5.51). An increase in caregiver’s educational status was associated with higher odds of uptake of HIV testing among the children. Children whose caregivers had been tested for HIV were 8.3 times more likely to have been tested (AOR = 8.31, 95%CI = 5.67–12.19) compared to those whose caregivers have never been tested (Table 2).

## Discussion

Only 14.2% (568) of a total 4037 children and young adolescents who participated in this study have ever been tested for HIV. This is slightly lower compared with result from a population-based survey done in Kenya where 16.4% of children less than 15 years had ever tested for HIV [25]. Contrary to result from this study, more children (34.7%) in a facility based study done in Cameroon had previously been tested for HIV [26]. In achieving population-level viral suppression and epidemic control in a state with the highest prevalence of HIV in Nigeria, a widespread coverage through targeted HIV testing is essential [20]. Although testing for HIV is the cornerstone of ultimate prevention, most children and young adolescents are still unaware of their status [27]. Testing uptake is higher among adults compared to children as evidenced from the 2017 Multiple Indicators Cluster survey [28]. There is evidence that HIV negative women seroconvert during the breast feeding period [29, 30]. These children are usually not considered exposed because their mothers were negative during antenatal resulting in missed opportunities. While there is evidence of mother to child transmission due to the high prevalence of HIV in Akwa-Ibom state [7], testing of exposed children has not been optimal [2]. Risky sexual behavior among young adolescents also make them vulnerable to HIV infection. This low prevalence of testing uptake can be attributed to less autonomy and decision-making power including health-seeking behaviour associated with this age group. It further buttresses the lack of evidence for HIV Testing Service approaches targeted towards children and adolescents [31]. The study sample is made up of 50.8% male (2048) and 49.2% females (1989). The ratio of males to females is about 1:1 which indicates that approximately, equal number of males and females were involved in the study. This is similar to the census held in Nigeria in 2006 [32]. The study was done on children and young adolescent from 0 to 14 years because Akwa-Ibom has the highest prevalence of HIV in Nigeria [7] with an increased risk of mother-to-child transmission. Also, the increase in risky behaviour associated with adolescents globally [33], makes it necessary to determine if they have ever been tested as well as understanding the correlates of testing uptake among them.

The mean age of the children and adolescents was 6.20 ± 3.91. Result showed approximately 15.0% of children less than 10 years and only 10.4% of adolescents aged 10-14 years have ever been tested for HIV. Although adolescents are at a greater risk of being infected, the testing rate is still low. In a similar study to assess the prevalence and correlates of HIV testing in Uganda, it was discovered that only 10.7% of adolescents aged 10–14 years had ever been tested for HIV [15]. Results of this study showed sex and age of the children and adolescents were not significantly associated with uptake of HIV testing. This was found to be consistent with a study from Western Kenya which showed the sex and age of a child had no significant association with acceptance of HIV testing [34].

Despite the higher percentage of children and young adolescent in urban area (18.3% vs. 12.2%) ever testing for HIV, location was not significantly associated with uptake of HIV testing in this study. This was inconsistent with a facility based cross-sectional study in Tanzania where mothers or caregivers who resided in the urban region were four times more likely to send their children for HIV test compared to their counterparts in the rural communities [35]. The result from this study may be due to the presence of primary health care facilities and community health care workers in both rural and urban parts of Akwa-Ibom.

Although more children enrolled in school had been tested for HIV, this was not significant in the study. However, Ssebunya et al. reported that adolescents with higher educational status were more likely to have been tested for HIV [15]. The study by Ssebunya et al. included adolescents up to 19 years who needed no parental consent before getting tested for HIV [15]. Although less common, transfusion of contaminated blood remains a very important cause of paediatric HIV infection in developing countries [36]. However, due to the screening of donated blood for evidence of HIV infection, the risk of acquiring HIV from blood transfusions is extremely low. From the result, 46.1% of children and young adolescents who have been transfused have been tested. This was found to be a significant correlate of uptake of HIV testing. Those who have been transfused were 5.3 times more likely to have been tested. This can be explained with the fact that blood recipients are also screened for HIV before being transfused due to medico-legal reasons.

Age as well as the sex of the caregiver were not significant in the multiple logistic regression model. Contrary to the result of this study, Makau, Okwara & Oyore in 2015 discovered that caregiver’s age was a significant predictor of testing uptake [37]. A similar study done in Cameroon identified the sex of a caregiver as a significant correlate as women were more likely to take their children for HIV testing services [26]. This could be due to the study being facility-based considering the feminization of uptake of HIV services in Cameroon [26, 38]. The association between caregivers’ educational level and testing uptake is consistent with other studies. Children of caregivers with no education had less chance to have been tested compared to children of caregivers with tertiary education. Higher education attainment was a strong correlate of testing uptake, which is in accordance with positive role of education in promoting general health status [16]. This finding suggests that educated caregivers may be more knowledgeable about the risk of HIV and understand the benefits of testing for HIV in both children and young adolescents. This finding reaffirms the results from Zambia showing that caregivers with higher level of education were likely to be more knowledgeable about mother-to-child transmission [39] and will be more willing to present their children for testing. In a cross sectional facility based study done in Tanzania, higher chances to access testing services were observed among children whose caregivers had attained high education level [40].

Caregivers’ knowledge of HIV was not significant in the uptake of HIV testing in children and young adolescents. This might be due to the high level of stigmatization associated with HIV. Despite the adequate knowledge of HIV, caregivers might prefer to remain unaware of the status of their children for fear of stigmatization if the result turns out positive. Contrary to results from this study, Makau et al. reported in their study on factors influencing uptake of EID among mothers in Kenya that good maternal knowledge is a significant predictor of EID uptake [37]. Samson et al. also discovered a significant association as having adequate knowledge was associated with an increase in uptake of HIV testing [35]. Results indicates that 23.5% of children whose caregivers knew their status have been tested for HIV. This was significant as those who had been previously tested were 8.3 times more likely to have been tested for HIV compared to those who have not. Vreeman et al. in 2010 stated that children were less likely to be tested for HIV if their mother/caregiver had an unknown HIV status [34]. According to Bwana et al., a mother’s ignorance of her status is a significant predictor of missed opportunity to access HIV testing services [40]. Children of HIV infected mothers/caregiver were more likely to get tested due to the knowledge they have acquired about HIV and the willingness to prevent their children from contracting the virus.

The limitation of AKAIS is mainly the cross-sectional nature of the survey, which only provides a snapshot of the HIV prevalence in Akwa Ibom state. The generalizability of the study findings is limited to the region under study. The study only assessed information on children and young adolescents aged 0-14 years, therefore, the findings may not be generalizable to adolescents above 14 years. However, the large sample size of 4037 among children aged 0–14 years helps provide important insights about HIV testing in children. Collecting accurate information about sensitive topics is difficult, particularly topics pertaining to HIV testing. Respondents may have provided socially desirable answers rather than provide answers that accurately reflect their experiences. Respondents may not readily disclose whether they have previously tested for HIV, for fear of being identified as HIV positive.

## Conclusion

This study has demonstrated that uptake of HIV testing among children and young adolescents in Akwa-Ibom state is low. There is a need for HIV interventions to be targeted towards this age group. Scaling up HIV testing services through provider initiated testing and counseling and family index testing will help increase the uptake of testing services among children and young adolescents. Addressing the knowledge gap amongst caregivers especially in rural areas is crucial towards improving the effectiveness of HIV interventions. Community-based health education and testing will also increase access to HIV services. This will have a positive effect towards ending the AIDS epidemic by 2030.

## Supplementary Information

**Additional file 1.** AKAIS Adult Individual Questionnaire. Questions pertaining to children 0 months–14 years.

**Additional file 2.** AKAIS Adolescent Individual Questionnaire (10-14 years). Questions on demographic characteristics, HIV knowledge, attitudes, risk perception and HIV testing for adolescents between 10 and 14 years. Questions about sexual activity for adolescents aged 12–14 years.

## Data Availability

The datasets used and analyzed in this study are available from the corresponding author upon request.

## Abbreviations

AIDS: Acquired Immunodeficiency Syndrome
AKAIS: Akwa-Ibom AIDS Indicator Survey (AKAIS)
AOR: Adjusted odds ratio
ARV: Anti-retroviral
CDC: Centres for Disease Control and Prevention
CI: Confidence interval
COR: Crude odd ratio
EA: Enumeration area
EGPAF: Elizabeth Glaser Pediatric AIDS Foundation
EID: Early infant diagnosis
HIA: HIV Impact Assessment
HIV: Human Immunodeficiency Virus
LGA: Local Government Area
NAIIS: Nigeria HIV/AIDS Indicator and Impact Survey
PMTCT: Prevention of Mother-to-Child Transmission
PPS: Probability proportional to size
SVY: Survey
UNAIDS: Joint United Nations Programme on HIV/AIDS
